# Draft Whole-Genome Sequences of Seven Isolates of Klebsiella pneumoniae from New Zealand Sea Lions

**DOI:** 10.1128/MRA.01270-18

**Published:** 2018-11-21

**Authors:** Komkiew Pinpimai, Wendi D. Roe, Patrick J. Biggs, Keren E. Dittmer

**Affiliations:** a Pathobiology Group, School of Veterinary Science, Massey University, Palmerston North, New Zealand; b ^*m*^EpiLab, Infectious Disease Research Centre, School of Veterinary Science, Massey University, Palmerston North, New Zealand; Indiana University Bloomington

## Abstract

Klebsiella pneumoniae is a Gram-negative bacterium that may cause infection in a broad range of hosts. We report here the genome sequences of seven K. pneumoniae isolates from New Zealand sea lions.

## ANNOUNCEMENT

Klebsiella pneumoniae is an opportunistic bacterium causing nosocomial and community-acquired infections. In the 1980s, an invasive form (hypervirulent strain) of K. pneumoniae infection causing primary liver abscesses and septicemia was described (1, 2). Most of the isolates had a hypermucoviscous phenotype (positive string test) and possessed *rmpA* and *rmpA2* genes (1, 3). This hypermucoviscous phenotype has since been reported in animals (4–8).

K. pneumoniae caused mass mortality events in New Zealand sea lion (NZSL) pups in the 2001–2002 and 2002–2003 breeding seasons at Sandy Bay, Subantarctic Enderby Island, New Zealand (9). After these mass mortality events, K. pneumoniae became endemic in this population (10).

Approval for the sampling of live animals was obtained from the New Zealand Department of Conservation Animal Ethics Committee (approval identification numbers AEC52, AEC86, AEC157, AEC158, AEC174, AEC200, and AEC232). K. pneumoniae was isolated from tissues and swabs (Table 1) using MacConkey agar, incubated at 37°C in aerobic conditions. In this study, seven K. pneumoniae isolates from NZSLs were whole-genome sequenced. A NucleoSpin soil kit (Macherey-Nagel, GmbH & Co. KG, Düren, Germany) was used to extract genome-quality DNA from a single colony, which was sent to New Zealand Genomics Limited (Massey Genome Service, Massey University, Palmerston North, New Zealand). A fragment library was prepared using an Illumina TruSeq DNA library preparation kit (version 1) (Illumina, Inc., Scorsby, Victoria, Australia). Paired-end reads (2 × 250 bp) were obtained from a MiSeq instrument (Illumina, Inc., San Diego, CA, USA) and were subject to quality control (Cutadapt, FastQC, and SolexaQA++). The isolates were *de novo* assembled using SPAdes (version 3.10) (11). The contigs of each isolate produced from SPAdes were annotated by Prokka (version 1.1.2) (12). The sequence type (ST) and serotype of each bacterial isolate were determined using the Bacterial Isolate Genome Sequence Database (BIGSdb) server (http://bigsdb.pasteur.fr/klebsiella/klebsiella.html). Virulence genes were identified by mapping the reads of each isolate along with 1,000-bp flanks on either side of each virulence gene sequence. Genome sizes, numbers of contigs, STs, sources, serotypes, and virulence genes are summarized in Table 1.

**TABLE 1:** Description of *K. pneumoniae* strains sequenced, their genomic characteristics, and associated virulence factors

Isolate	GenBank accession no.	SRA accession no.	Source	Location	Host status	Tissue sample	Serotype	ST	String test result^*a*^	Length (bp)	No. of reads	No. of contigs	% GC content	Virulence genes
E02_03_112Ph	QVFM00000000	SRR7657834	NZSL pup	Enderby Island	Fatal infection	Brain	K2	86	+	5,662,712	4,336,820	730	56.2	*rmpA*, *wabG*, *uge*, *iroN*, *irp2*, *iucD*, *iutA*, *ybtS*, *mrkD*
E11_12_24Ph	QVFN00000000	SRR7657833	NZSL pup	Enderby Island	Fatal infection	Atlanto-occipital joint swab	K2	86	+	5,672,644	4,514,658	799	56.3	*rmpA*, *wabG*, *uge*, *iroN*, *irp2*, *ybtS*, *mrkD*
S13_04Ph	QVFO00000000	SRR7657836	NZSL pup	Otago region	Fatal infection	Joint fluid swab	K2	86	+	5,611,619	4,851,180	624	56.4	*rmpA*, *wabG*, *uge*, *iroN*, *irp2*, *iucD*, *iutA*, *ybtS*, *mrkD*
D14_15_08Ph	QVFP00000000	SRR7657835	NZSL pup	Dundas Island	Fatal infection	Brain	K2	86	+	5,353,362	2,209,303	81	57.5	*rmpA*, *wabG*, *uge*, *iroN*, *irp2 ybtS*, *mrkD*
C14_15_09Ph	QVFQ00000000	SRR7657838	NZSL pup	Campbell Island	Carrier	Brain	Non-K1/1K2	2843	+	5,644,618	2,250,661	105	57.1	*wabG*, *uge*, *iroN*, *irp2*, *iucD*, *iutA*, *ybtS*, *mrkD*
E09_10_13Ph	QVFR00000000	SRR7657837	NZSL adult	Enderby Island	Carrier	Tracheobronchial lymph nodes	Non-K1/1K2	2843	−	5.703,228	2,358,085	97	57.0	*wabG*, *uge*, *iroN*, *irp2*, *iucD*, *iutA*, *ybtS*, *mrkD*
C14_9476Ph	QVFS00000000	SRR7657839	NZSL adult	Campbell Island	Carrier	Rectal swab	K2	86	+	5,324,629	1,645,412	93	57.5	*rmpA*, *wabG*, *uge*, *iroN*, *irp2*, *ybtS*, *mrkD*

a +, positive; −, negative.

Aerobactin, an iron-chelating compound, is suggested to enhance virulence in hypervirulent K. pneumoniae strains (13). However, in this study, two isolates from sea lion pups with fatal K. pneumoniae infections lacked the genes *iucD* and *iutA* that code for aerobactin, suggesting that other factors may play roles in their pathogenicity. Another study suggested a relationship between yersiniabactin (encoded by the *ybt*, *irp*, and *fyu* genes) and hypervirulence (14). In our study, *ybts* and *irp2* were found in isolates from both animals with fatal infections and healthy carriers, suggesting that yersiniabactin was not specific for hypervirulence. More studies are needed to clarify the association between chelating compounds and virulence in K. pneumoniae.

One isolate, with a positive string test, did not possess the *rmpA* and *rmpA2* genes considered to play a key role in the hypermucoviscous phenotype (15), suggesting that other factors may contribute to the expression of the hypermucoviscous phenotype (16).

This is the first report of draft whole-genome sequences of K. pneumoniae isolated from NZSLs. The data from this study will provide further information to help understand the genomic relationships of the K. pneumoniae strains that circulate in NZSLs.

### Data availability.

The draft whole-genome shotgun sequences described here have been deposited in DDBJ/ENA/GenBank under the accession numbers listed in Table 1. Raw sequence reads have been deposited in the NCBI Sequence Read Archive under the accession numbers listed in Table 1.
